# Comparison of trastuzumab emtansine, trastuzumab deruxtecan, and disitamab vedotin in a multiresistant HER2-positive breast cancer lung metastasis model

**DOI:** 10.1007/s10585-024-10278-2

**Published:** 2024-02-17

**Authors:** Negar Pourjamal, Narjes Yazdi, Aleksi Halme, Vadim Le Joncour, Pirjo Laakkonen, Pipsa Saharinen, Heikki Joensuu, Mark Barok

**Affiliations:** 1 Helsinki University Hospital and University of Helsinki, Helsinki, Finland; 2 Laboratory of Molecular Oncology, University of Helsinki, Helsinki, Finland; 3 Translational Cancer Medicine Research Program, Faculty of Medicine, University of Helsinki, Helsinki, Finland; 4 Neuroscience Center, Helsinki Institute of Life Sciences (HiLIFE), University of Helsinki, Helsinki, Finland; 5 Laboratory Animal Center, Helsinki Institute of Life Science (HiLIFE), University of Helsinki, Helsinki, Finland; 6 Wihuri Research Institute, University of Helsinki, Helsinki, Finland; 7 Department of Oncology, Helsinki University Hospital and University of Helsinki, Helsinki, Finland; 8 Biomedicum Helsinki, Haartmaninkatu 8, Helsinki, 00290 Finland

**Keywords:** Breast cancer, Human epidermal growth factor receptor 2, Trastuzumab emtansine, Trastuzumab deruxtecan, Disitamab vedotin, Antibody drug conjugate, Lung metastasis

## Abstract

Human epidermal growth factor 2 (HER2)-positive breast cancer with lung metastases resistant to targeted agents is a common therapeutic challenge. Absence of preclinical lung metastasis models that are resistant to multiple anti-HER2 targeted drugs hampers the development of novel therapies. We established a novel HER2-positive breast cancer cell line (L-JIMT-1) with a high propensity to form lung metastases from the parenteral JIMT-1 cell line by injecting JIMT-1 cells into immunodeficient SCID mice. Lung metastases developed in all mice injected with L-JIMT-1 cells, and more rapidly and in greater numbers compared with the parental JIMT-1 cells. L-JIMT-1 cells expressed more epidermal growth factor receptor and HER2 than JIMT-1 cells. L-JIMT-1 cells were resistant to all five tyrosine kinase inhibitors tested in vitro (afatinib, erlotinib, lapatinib, sapitinib, and tucatinib). When we compared JIMT-1 and L-JIMT-1 sensitivity to three HER2-targeting antibody-drug conjugates (ADCs) trastuzumab emtansine (T-DM1), trastuzumab deruxtecan (T-DXd), and disitamab vedotin (DV) in vitro, JIMT-1 cells were resistant T-DXd, partially sensitive to T-DM1, and sensitive to DV, while L-JIMT-1 cells were resistant to both T-DM1 and T-DXd, but moderately sensitive to DV. In a mouse model, all three ADCs inhibited the growth of L-JIMT-1 lung metastases compared to a vehicle, but DV and T-DXd more strongly than T-DM1, and DV treatment led to the smallest tumor burden. The L-JIMT breast cancer lung metastasis model developed may be useful in the evaluation of anti-cancer agents for multiresistant HER2-positive advanced breast cancer.

**Supplementary Information:**

The online version contains supplementary material available at 10.1007/s10585-024-10278-2.

## Introduction

About 15–20% of breast cancers overexpress the human epidermal growth factor receptor 2 (HER2), which is the key therapeutic target for patients with HER2-positive cancer [1, 2]. Several drugs targeting HER2 have been approved for the treatment of HER2-positive breast cancer including monoclonal antibodies, tyrosine kinase inhibitors (TKIs), and antibody-drug conjugates (ADCs) [3]. Most patients with advanced HER2-positive breast cancer achieve a durable response when treated with these agents, but emergence of drug resistance is common [3–5]. Patients with advanced breast cancer with low HER2 expression may also benefit from the treatment with the HER2 targeting ADC trastuzumab deruxtecan (T-DXd) [6].

HER2 overexpressing breast cancers frequently give rise to visceral metastases located in the lung and in the liver. The lung is the first site of distant metastases in up to 25% of the patients with HER2-positive breast cancer, and lung metastases are more common in the patient population with HER2-positive breast cancer compared to the other main biological subgroups of breast cancer [7, 8]. Lung metastases are often symptomatic and may be associated with persistent cough, dyspnea, pain, and accumulation of pleural fluid.

Progressing HER2-positive breast cancer metastatic to the lung and resistant to several HER2-targeted agents is a common therapeutic challenge in the clinic. To the best of our knowledge, there are no established animal models available for such breast cancers. This hampers the development of therapies for patients with HER2-positive breast cancer with drug-resistant lung metastases, since preclinical models can be indispensable not only for comparisons of the approved anti-HER2 targeted agents, but for the development of novel agents and novel drug combinations as well. We describe and characterize here a novel mouse model of HER2-positive breast cancer metastatic to the lungs that shows resistance to multiple frequently used anti-HER2 agents, thus reflecting a common clinical scenario.

We found that JIMT-1 breast cancer cells frequently give rise to lung metastases. JIMT-1 cell line is resistant to anti-HER2 monoclonal antibody drugs trastuzumab and pertuzumab, and the orally administered HER2-targeting TKI lapatinib [9, 10]. We derived a new cell line from the JIMT-1 cells, called L-JIMT-1 (lung metastasis JIMT-1) that has an even higher propensity to give rise to lung metastases than the parental JIMT-1. L-JIMT-1 turned out to be resistant to multiple drugs and does not respond to several anti-HER2 TKIs, and it responds poorly also to the approved anti-HER2 ADCs trastuzumab emtansine (T-DM1) and T-DXd. Interestingly, a novel anti-HER2 ADC, disitamab vedotin (DV), turned out to be more effective than T-DM1 or T-DXd in the treatment of mice with L-JIMT-1 lung metastases. Besides describing a novel lung metastasis model, to our knowledge, this is the first study that directly compares the anti-cancer efficacy of T-DM1, T-DXd, and DV.

## Methods

### Ethical aspects

The Committee for animal experiments of the District of Southern Finland approved the animal experiments under the licenses ESAVI/403/2019 (valid 1.4.2019–31.3.2022), ESAVI/11614/2022 (valid 1.4.2022–30.8.2022), and ESAVI/10262/2022 (valid 4.5.2022–4.5.2025). The reporting in the manuscript follows the recommendations in the ARRIVE guidelines (https://arriveguidelines.org/arrive-guidelines).

### Cell lines

The HER2-positive human breast cancer cell line JIMT-1 was obtained from the Laboratory of Cancer Biology, University of Tampere (Tampere, Finland; also available via DSMZ-German Collection of Microorganisms and Cell Cultures GmbH, Leibniz Institute, Braunschweig, Germany). The HER2-negative human breast cancer cell line MCF-7 and the HER2-positive human breast cancer cell line SKBR-3 were from the American Type Culture Collection (ATCC, Manassas, VA, USA). The cell lines were cultured according to the recommended specifications. The cell lines were mycoplasma-free. Authentication of the cell lines was performed using a short tandem repeat analysis (JIMT-1, October 2018 and February 2023; MCF-7, October 2018 and March 2023; L-JIMT-1, March 2023; SKBR-3, March 2023).

### JIMT-1 lung metastasis model

To investigate the metastasis sites of JIMT-1 cells, 1 × 10^5^ human JIMT-1 breast cancer cells in 100 µL phosphate buffered saline (PBS) were injected into the tail vein of five to eight-week-old female SCID mice (C.B-17/IcrHan Hsd-Prkdcscid, Envigo RMS B.V., Horst, The Netherlands).

For the xenograft studies, five to eight-week-old female SCID mice were injected subcutaneously with 2 × 10^6^ JIMT-1 or L-JIMT-1 cells in 100 µL of the cell culture medium, or with 2 × 10^6^ JIMT-1 or L-JIMT-1 cells in 50 µL of the cell culture medium into the left mammary fat pad.

The general condition of the mice was assessed by visual inspection, longitudinal weight measurements, and by assessing the body condition score [11]. Euthanasia was performed if significant weight loss, breathing difficulty, or deterioration of the body condition score occurred, or, in case of the xenografts, if any tumor dimension exceeded 15 mm or tumor ulceration occurred. Mice were sacrificed using CO_2_ inhalations followed by cervical dislocation.

### Establishment of the L-JIMT-1 cell line and a mouse model

**Fig. 1:**
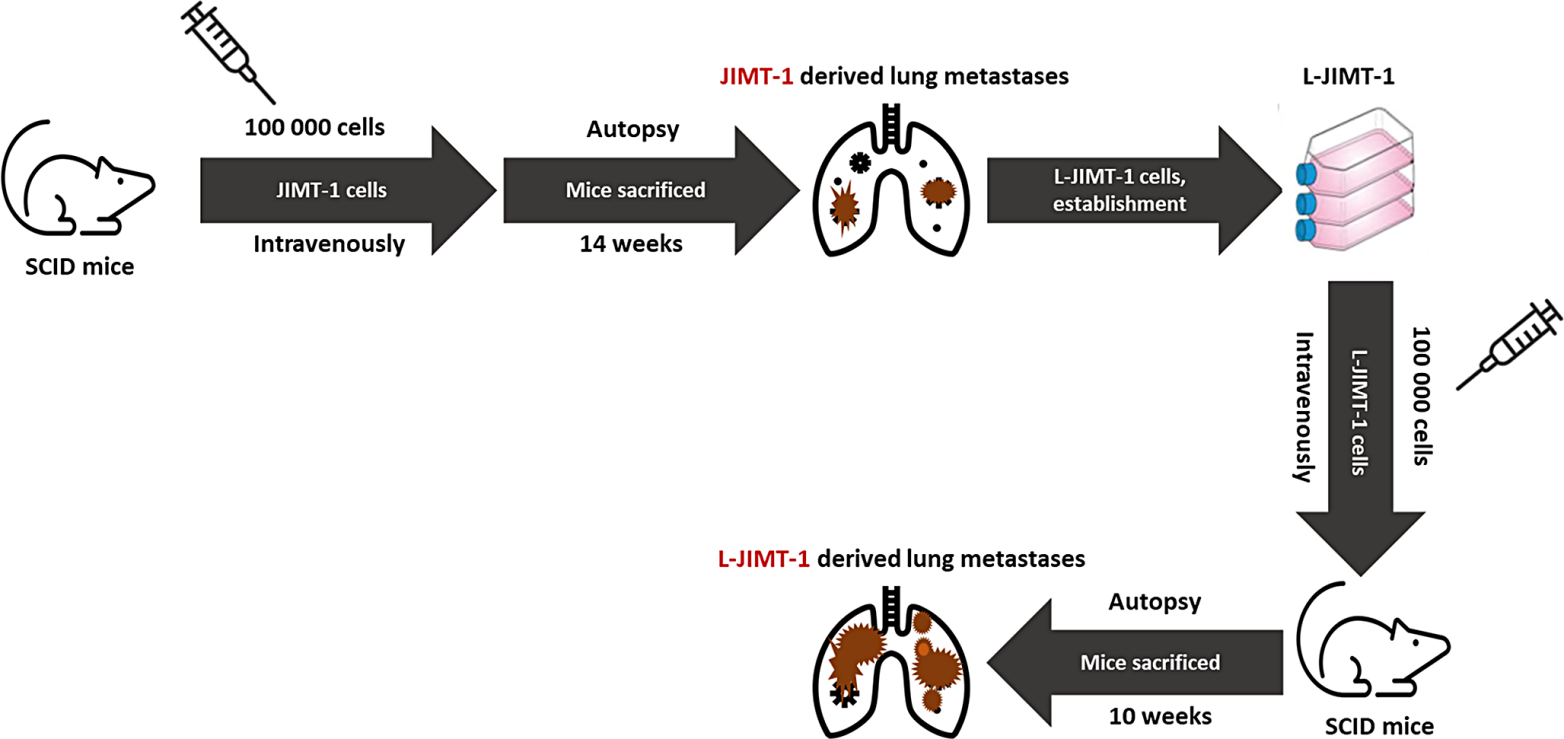
Establishment of a novel HER2-positive breast cancer lung metastasis model. The L-JIMT-1 cell line was established from a lung metastasis of a SCID mouse injected intravenously with the JIMT-1 cells. When the L-JIMT-1 cells were injected intravenously into mice, more lung metastases developed within a shorter time compared to the JIMT-1 injected mice

The L-JIMT-1 cell line was derived from a lung metastasis that developed about 3 months after the injection of JIMT-1 cells into the tail vein of SCID mice (Fig. 1). After euthanasia, a lung metastasis was removed and cut into small pieces of about 1 mm^2^ in size with a sterile blade, washed twice with sterile PBS, and placed in a culture dish containing Dulbecco´s Modified Eagle Medium (DMEM) supplemented with 10% heat-inactivated fetal bovine serum (FBS) and penicillin. After 3 days, debris and dead cells were removed, and the medium was replaced. Confluent cultures were trypsinized and split using the same cell culture medium. To generate L-JIMT-1 metastases, 1 × 10^5^ L-JIMT-1 cells in 100 µL PBS were injected into the tail vein of five to 8-week-old female SCID mice, following which the mice were observed and euthanized as described above.

### Immunohistochemistry

The lungs, the liver, the kidneys, and the spleen were collected and fixed in 4% buffered formaldehyde for 24 h, processed into paraffin, and sectioned. For immunohistochemistry, 15 4-µm thick subserial sections were cut from the liver, the kidney, and the spleen, and from both the costal and medial surfaces of the lungs. The sections were deparaffinized followed by antigen-retrieval in a sodium citrate buffer (10 mM Sodium Citrate, pH 6.0) using a 2100 Antigen Retriever (Aptum Biologics Ltd., Southampton, UK) following the manufacturer’s recommendations. After blocking for non-specific binding, the primary anti-HER2 antibody (SP3, Thermo Fisher Scientific, Waltham, MA, USA), anti-progesterone receptor (PgR) antibody (PGR-312, Leica Biosystems, Newcastle, UK), and anti-estrogen receptor (ER) antibody (6F11, Leica Biosystems) were applied at optimized concentrations and incubated overnight at 4 °C. The primary antibody binding was detected using a BrightVision Poly-HRP anti mouse kit (VWR, Radnor, USA) and 3,3′-diaminobenzidine (ImmPACT DAB, Vector Laboratories, Burlingame, CA, USA) following the manufacturer’s recommendations. The tissue sections were counterstained with hematoxylin. The stained slides were imaged with an 20x objective of an Olympus BX50 microscope (Olympus Corporation, Tokyo, Japan) integrated with a SlideStrider objective slide scanner (JILab Inc, Tampere, Finland) or with a 20x objective of a Zeiss Axio Scan.Z1 Slide Scanner (Carl Zeiss, Göttingen, Germany). The number of metastases were counted on the HER2-stained subserial sections, and the largest diameter of the lung metastases was measured with the ZEN 3.1 software (Carl Zeiss, Göttingen, Germany).

### Flow cytometry

Flow cytometry was performed using an Accuri C6 Flow Cytometer (Accuri Cytometers, Inc., Ann Arbor, MI, USA). To assess cell surface epidermal growth factor receptor (EGFR), HER2, and HER3 expression, the cells were trypsinized and washed with 1% bovine serum albumin (BSA) in PBS. EGFR, HER2, and HER3 receptors were labeled using AlexaFluor647-anti-human EGFR (352918, BioLegend, San Diego, CA, USA), AlexaFluor647-anti-human HER2 (324412, BioLegend), and APC-anti-human HER3 (324708, BioLegend) primary antibodies, respectively, for 30 min at 4 °C. The cells were then washed twice with PBS and fixed in 2% formaldehyde.

### Tumor volume measurement

Micro-CT imaging of the lungs was performed before the injection of L-JIMT-1 cells on week 0, and then bi-weekly from week 7 onwards with a Quantum GX2 microCT Imaging System (Perkin Elmer, Waltham, MA, USA). The mice were scanned under 2–3% isoflurane anesthesia. Images were acquired using respiratory gating and voltage 90 kV, current 80 µA, and a total scan time of 4 min. The images from week 0 and weeks 7, 9, 11, 13, and week 15 were analyzed using a 3D Slicer software [12]. Image analysis was performed using threshold segmentation to identify the anatomical structures. The noise was reduced, and the image quality was improved through the application of the median filter function of the 3D Slicer software. A suitable threshold value for the aerated lung segmentation was then determined and applied to all images of the same mouse at different time points. A segment for the total chest space was also created. The combined tumor and vasculature volume (TVV) was determined by subtracting the aerated functional lung volume from the total chest space volume, as tumor tissue and vascular tissue have similar gray scale values that cannot be separated with the method. However, the volume of the non-tumor vasculature remains relatively constant as the tumor volume increases over time [13].

### Comparison the efficacy of T-DM1, T-DXd, and DV in the L-JIMT mouse model

To compare the anti-tumor efficacy of T-DM1, T-DXd, and DV on L-JIMT-1 lung metastasis, five to eight-week-old female SCID mice were injected intravenously with 1 × 10^5^ of L-JIMT-1 human breast cancer cells in 100 µL PBS. The mice were randomized, stratifying based on the tumor and vasculature volume values calculated from the micro-CT images taken on week 7, into four treatment groups: T-DM1 (5 mg/kg), T-DXd (5 mg/kg), DV (5 mg/kg), or PBS. Each treatment was administered intravenously once on week 8.

### In vitro drug sensitivity assay

The compounds investigated are summarized in Supplementary Table 1. The effects of five TKIs: lapatinib (Sigma-Aldrich, St. Louis, MO, USA), erlotinib (InvivoChem, Libertyville, IL, USA), afatinib (InvivoChem) sapitinib, (InvivoChem), and tucatinib (InvivoChem), and three ADCs: T-DM1 (Kadcyla, Roche Ltd., Basel, Switzerland), T-DXd (Enhertu, AstraZeneca, Cambridge, UK), and DV (MedChemExpress, Monmouth Junction, NJ, USA) on cell growth were studied using the AlamarBlue method (Thermo Fisher Scientific) as described previously [14, 15]. Briefly, the cells were trypsinized and plated in flat-bottomed 96-well tissue culture plates. Afatinib, erlotinib, sapitinib, and tucatinib were tested at concentrations 0.1, 1, 10, 50, 100, 500, 1000, and 10,000 nMol/L, and lapatinib at 0.1, 0.6, 3, 16, 80, 400, 1000, and 2000 nMol/L, and the three ADCs at concentrations 0.0001, 0.0006, 0.003, 0.016, 0.08, 0.4, 1, 2, and 10 µg/mL. The numbers of viable cells were assessed after 5-day incubation, when the AlamarBlue reagent (Thermo Fisher Scientific) was added to the culture medium, and fluorescence was measured with a PHERAstar FS plate reader (BMG Labtech, Ortenberg, Germany) using excitation at 540 nm and emission at 590 nm. The fluorescence of the samples was normalized to the fluorescence of the cell-free culture medium. The results are presented as the percentage of viable cells relative to the non-treated control, obtained by dividing the fluorescence of the test samples by the fluorescence of the PBS-treated control. The drug concentration that resulted in half-maximal (50%) growth inhibition (IC_50_) was calculated using the Graphpad Prism 9 software (GraphPad Software, San Diego, CA, USA). The drug effect was assessed in three HER2-positive breast cancer cell lines (SKBR-3, JIMT-1, and L-JIMT-1) and in one control cell line (MCF-7) that does not harbor HER2 gene amplification and has low HER2 protein expression [16].

### Statistical analysis

The in vitro data are expressed as the mean ± standard deviation, and groups were compared using the Student’s t-test, ANOVA, and two-way repeated measures ANOVA. Non-normal distributions in the numbers of lung metastases between the JIMT-1 and L-JIMT-1 mouse groups were compared with the Mann-Whitney test. The times from the date of cancer cell injection to the date of euthanasia were compared between mouse groups with the Kaplan-Meier method and the log-rank test. Statistical calculations were carried out using the IBM SPSS version 24 (IBM, Armonk, NY, USA). All *p* values are 2-sided and not adjusted for multiple testing.

## Results

### JIMT-1 breast cancer cells give frequently rise to HER2-positive lung metastases

Weight loss and decrease in the body condition score occurred in 10 (83%) of the 12 SCID mice when followed up for a median of 15 weeks (range, 14 to 18 weeks) after intravenous injection of JIMT-1 cells. Macroscopic lung metastases were detected in all 10 mice at autopsy. When 15 sections were cut from the paraffin embedded lungs, presence of HER2-positive lung metastases could be confirmed in all 10 mice (median, 3 metastases/mouse; range, 0–16; Table 1). The metastases were negative for estrogen receptor (ER) and progesterone receptor (PgR) in immunostaining (Supplementary Fig. 1). No macroscopic metastases were found in the other organs investigated including the liver, the intestine, the spleen, the stomach, the kidneys, and the brain, and after subserial tissue sectioning, no metastases were found at immunohistochemistry in the liver, the kidneys, or the spleen. No metastases were found in the two remaining mice in any organ either at autopsy or at immunohistochemistry.

**Table 1:** Lung metastases in JIMT-1 and L-JIMT-1 mouse models

Cell type	No. of mice injected with cancer cells	No. of mice with lung metastases/total number of mice injected	Weeks from injection to euthanasia Median (range)	No. of lung metastases per mouse Median (range)	HER2 expression	Progesterone receptor expression	Estrogen receptor expression
JIMT-1	12	10/12 (83%)	15 (14, 18)	3 (0, 16)	Positive	Negative	Negative
L-JIMT-1	10	10/10 (100%)	10 (9, 14)*	12 (6, 18)**	Positive	Negative	Negative

*Abbreviations* HER2, human epidermal growth factor receptor 2; SCID, severe combined immunodeficiency

*p = 0.004 compared to JIMT-1 cells

**p < 0.001 compared to JIMT-1 cells

### L-JIMT-1 cells have a high propensity give rise to lung metastasis in SCID mice

We established the L-JIMT-1 cell line from a lung metastasis of a SCID mouse intravenously injected with the JIMT-1 cells (Fig. 1). After an intravenous injection with the L-JIMT-1 cells, weight loss and decrease in the body condition score occurred in all 10 SCID mice investigated already after a median observation time of 10 weeks (range, 9 to 14 weeks; Table 1). All mice had macroscopic lung metastases at autopsy, but no macroscopic or microscopic metastases were found in the other organs examined (the liver, the intestine, the spleen, the stomach, the kidney, and the brain). After cutting 15 subserial sections from the paraffin embedded lungs of each mouse, presence of HER2-positive, ER-negative, and PgR-negative lung metastases could be confirmed in all 10 mice (median, 12 metastases/mouse; range, 6–18; Table 1, Supplementary Fig. 1). Compared to the JIMT-1 mice, the L-JIMT-1 mice thus developed lung metastases more rapidly leading to earlier euthanasia (*p* < 0.001) and the L-JIMT-1 mice had a greater number of lung metastases (*p* = 0.004) (Table 1).

Both JIMT-1 and L-JIMT-1 cells formed tumors when injected subcutaneously or into the mammary fat pad of SCID mice, but no metastases were found at the autopsy (data not shown).

### L-JIMT-1 cells express more EGFR and HER2 than JIMT-1 cells

**Fig. 2:**
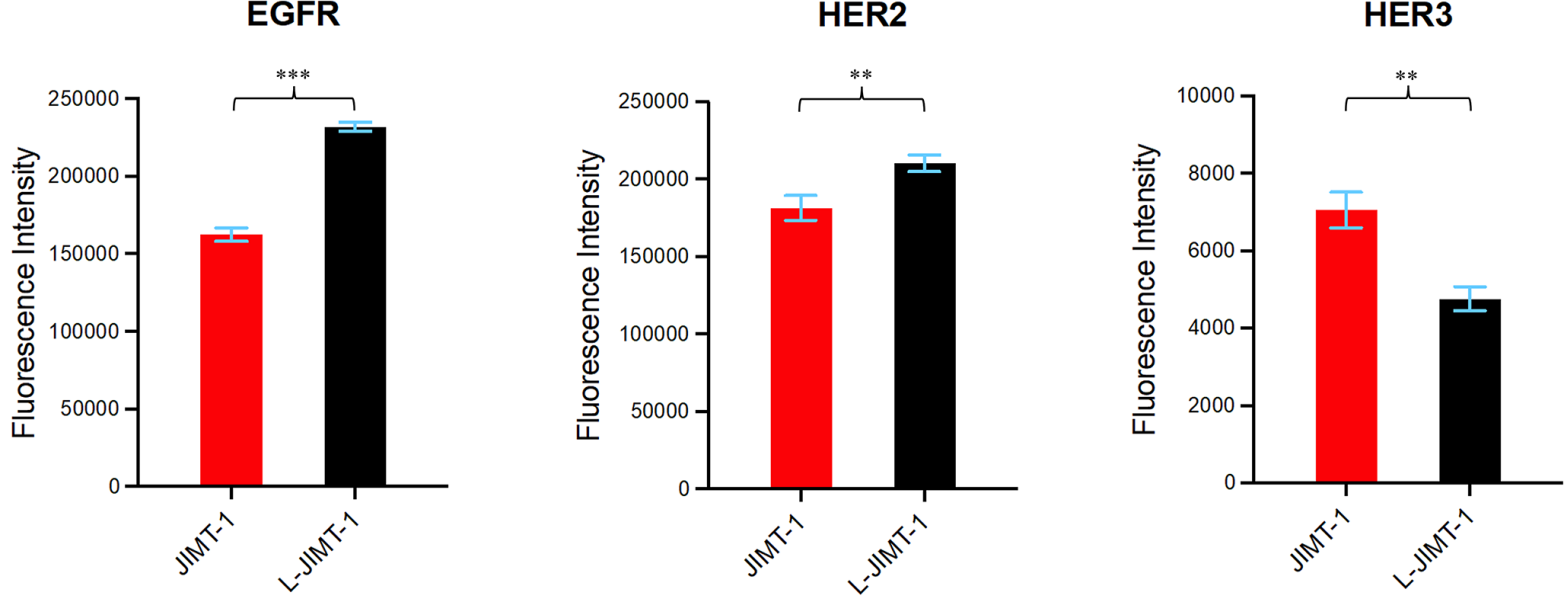
Flow cytometric quantification of cell surface EGFR, HER2, and HER3 expression on JIMT-1 and L-JIMT-1 cells. **p < 0.01, ***p < 0.001

EGFR and HER2 expression of JIMT-1 and L-JIMT-1 cell lines were investigated using flow cytometry. L-JIMT-1 cells had higher EGFR and HER2 protein expression (*p* < 0.001 and *p* = 0.006, respectively), but lower HER3 protein expression (*p* = 0.002) compared to the parental JIMT-1 cells (Fig. 2).

### L-JIMT-1 cells were insensitive to five small molecule EGFR/HER2 inhibitors in vitro

**Fig. 3:**
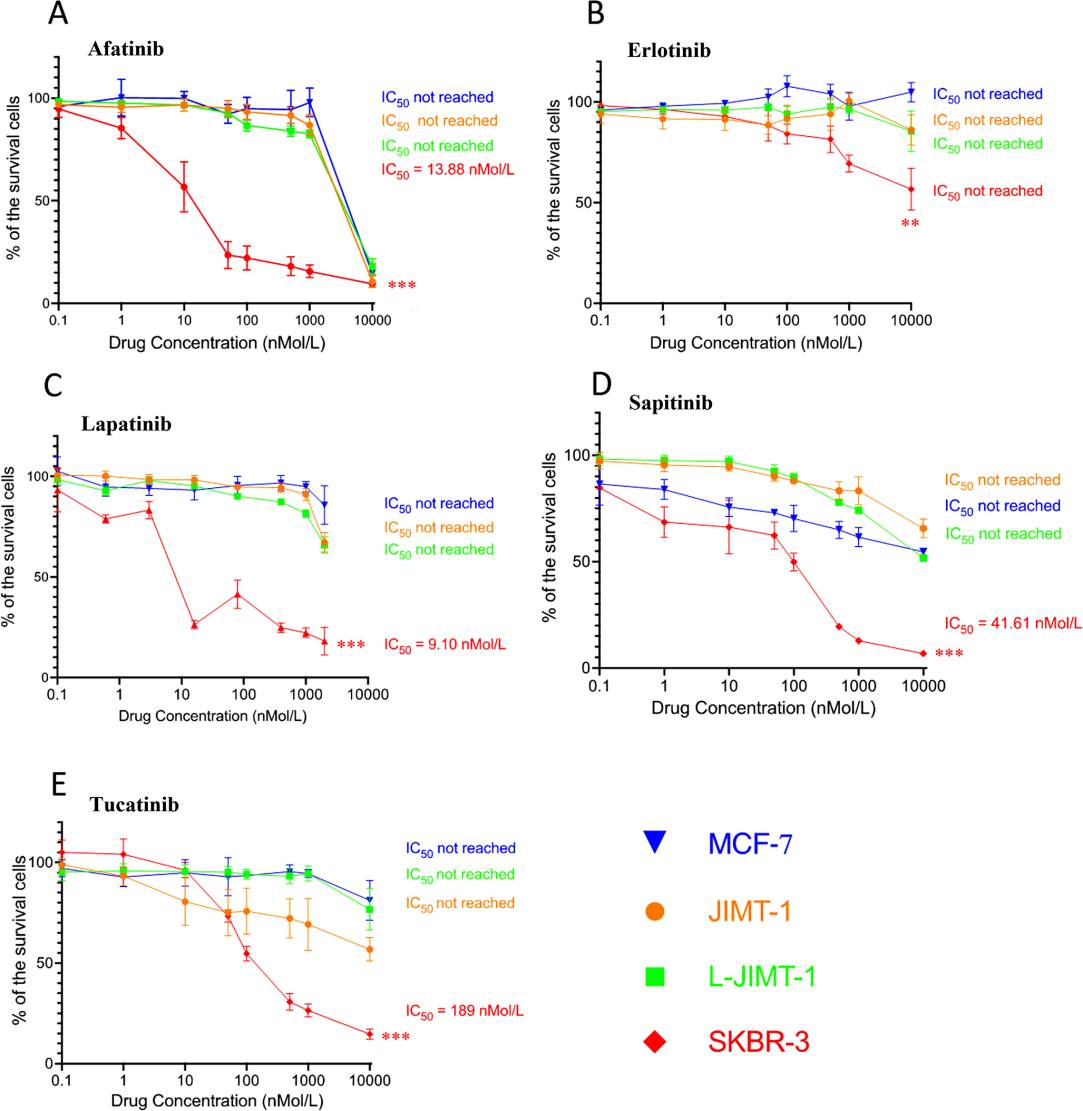
Growth inhibition of anti-EGFR and/or anti-HER2 tyrosine kinase inhibitors on JIMT-1 and L-JIMT-1 cells, and on MCF-7 (negative control) and SKBR-3 (positive control) cells (A-E). **p < 0.01, ***p < 0.001

We compared the growth-inhibitory effects of afatinib, erlotinib, lapatinib, tucatinib, and sapitinib that inhibit either EGFR, HER2, or both on JIMT-1 and L-JIMT-1 cells. The HER2-positive breast cancer cell line SKBR-3, known to be generally sensitive to HER2-targeting TKIs, was used as a positive control and the HER2-negative MCF-7 breast cancer cell line that is insensitive to anti-HER2 drugs as a negative control [16, 17]. Neither JIMT-1 nor L-JIMT-1 cells were sensitive to afatinib, erlotinib, lapatinib, or sapitinib (IC_50_ was not reached with any of the compounds). JIMT-1 cells tended to be more sensitive to tucatinib (IC_50_ not reached) than the L-JIMT-1 cells (*p* = 0.050) that were insensitive. As expected, MCF-7 cells were insensitive to all five TKIs and SKBR-3 cells were sensitive (the IC_50_ was reached except for erlotinib; Fig. 3).

### L-JIMT-1 cells were resistant to T-DM1 and T-DXd, but showed partial sensitivity to DV in vitro

**Fig. 4:**
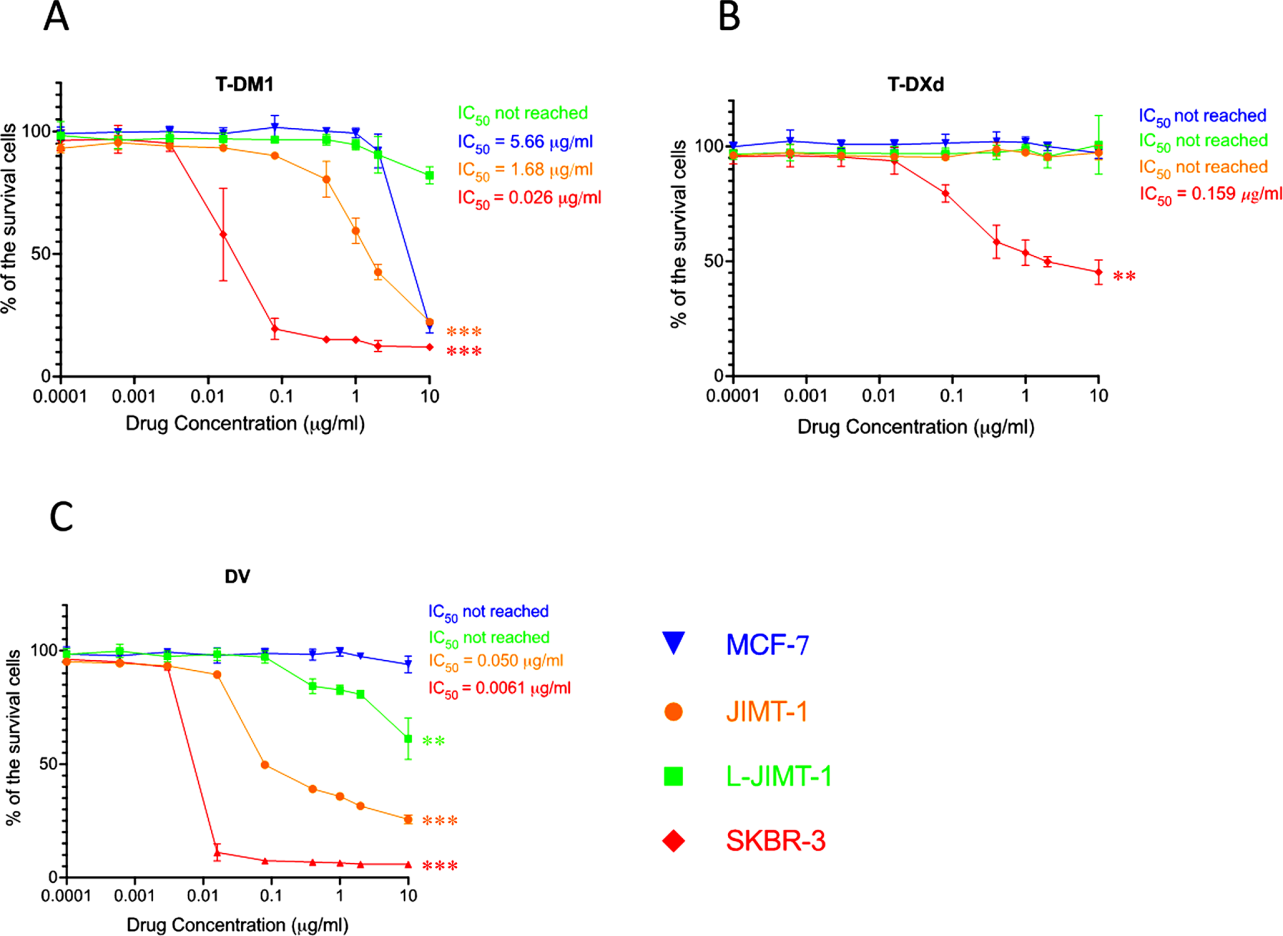
Growth inhibition of anti-HER2 antibody-drug conjugates on JIMT-1 and L-JIMT-1 cells, and on MCF-7 (negative control) and SKBR-3 (positive control) cells (A-C). **p < 0.01, ***p < 0.001

We next compared the growth-inhibitory efficacy of the HER2-targeting ADCs T-DM1, T-DXd, and DV on JIMT-1 and L-JIMT-1 cells in vitro. T-DM1 and DV inhibited the growth of JIMT-1 cells dose dependently, both achieving the IC_50_ concentration, whereas T-DXd lacked efficacy. On the JIMT-1 cells, DV was most effective of the three ADCs showing 33.6-fold greater potency than T-DM1 (Fig. 4).

L-JIMT1 cells were more resistant than JIMT-1 cells to T-DM1 and DV, and L-JIMT-1 cells were unresponsive to T-DM1 and T-DXd at all drug concentrations studied. L-JIMT-1 cells showed some sensitivity to DV at higher concentrations, but the IC_50_ was not reached. All three ADCs inhibited the growth of the positive control cells (SKBR-3) in a dose-dependent manner, each achieving IC_50_, but DV had 4.3-fold greater potency than T-DM1 and 26.1-fold greater potency than T-DXd in SKBR-3 cells. None of the ADCs inhibited the growth of MCF-7 cells (Fig. 4).

### The anti-cancer efficacy of the ADCs varied in an in vivo L-JIMT-1 lung metastasis model

**Fig. 5:**
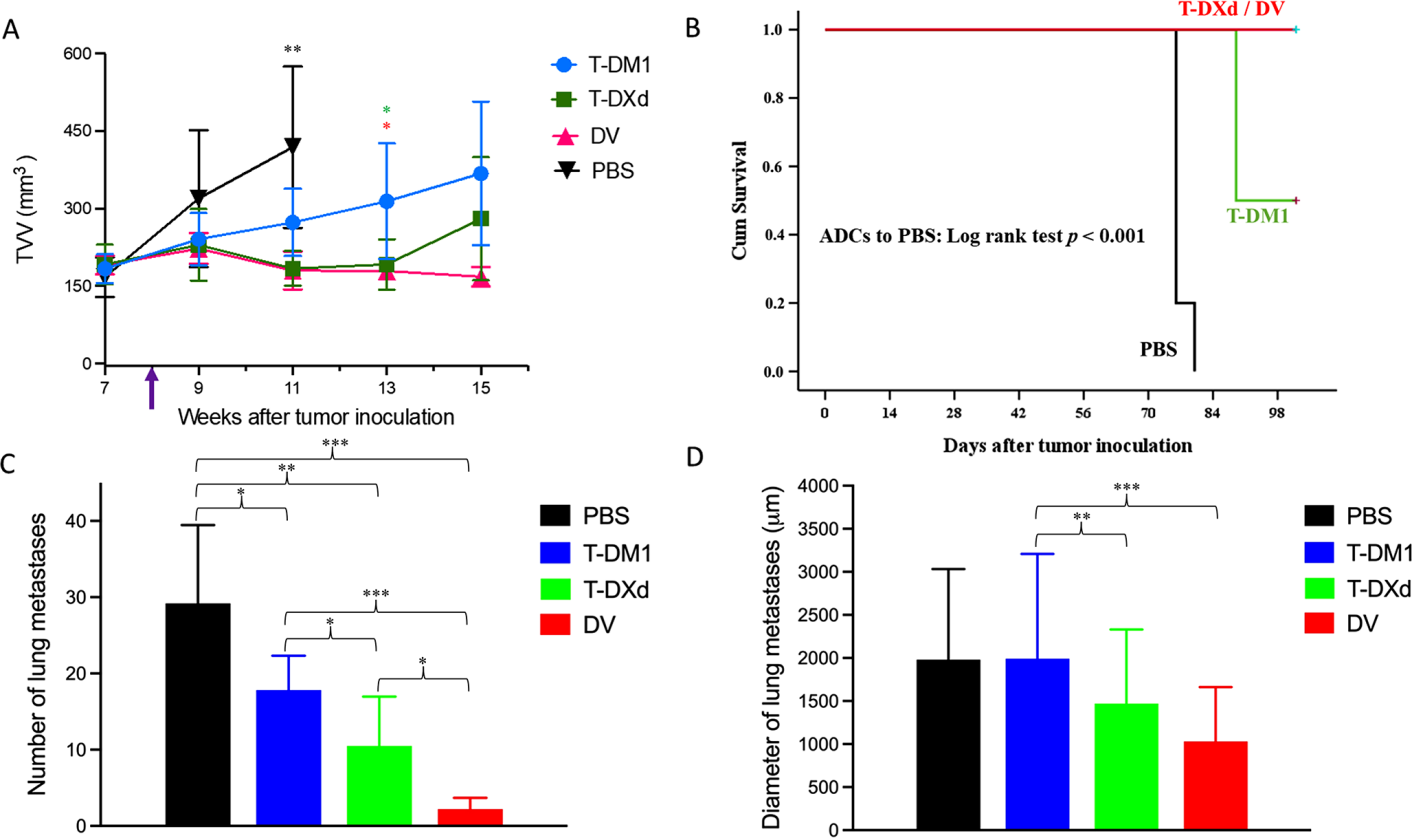
The effect of T-DM1, T-DXd, and DV on the growth of HER2-positive breast cancer lung metastases in SCID mice. **A**, Tumor and vasculature volumes (TVV) were assessed with bi-weekly micro-CT imaging. The mice received the treatment on week 8 (arrow). **B**, Survival of the mice. **C**, Number of lung metastases counted on subserial sections of the lungs. **D**, Largest diameter of lung metastases measured on subserial sections of the lungs. *p < 0.05, **p < 0.01, ***p < 0.001

Next, the efficacy of T-DM1, T-DXd, and DV was compared in an in vivo L-JIMT-1 lung metastasis model. Twenty-two SCID mice were injected intravenously with L-JIMT-1 cells, and 8 weeks after the injection the mice were split into four groups that were treated once with vehicle (PBS, *n* = 5) or with 5 mg/kg of either T-DM1 (*n* = 6), T-DXd (*n* = 6), or DV (*n* = 5). The mice were followed up longitudinally with bi-weekly tumor and vasculature volume measurements using a micro-CT. The three ADCs inhibited the growth of the lung metastases compared to the PBS control group by study week 11 (PBS versus all 3 ADCs, *p* = 0.001; Fig. 5A). By week 13, the T-DXd group and the DV-group had smaller tumor and vasculature volumes compared with the T-DM1-group (*p* = 0.048 and 0.020, respectively; the difference between the T-DXd and DV groups was not significant with *p* = 0.339).

**Fig. 6:**
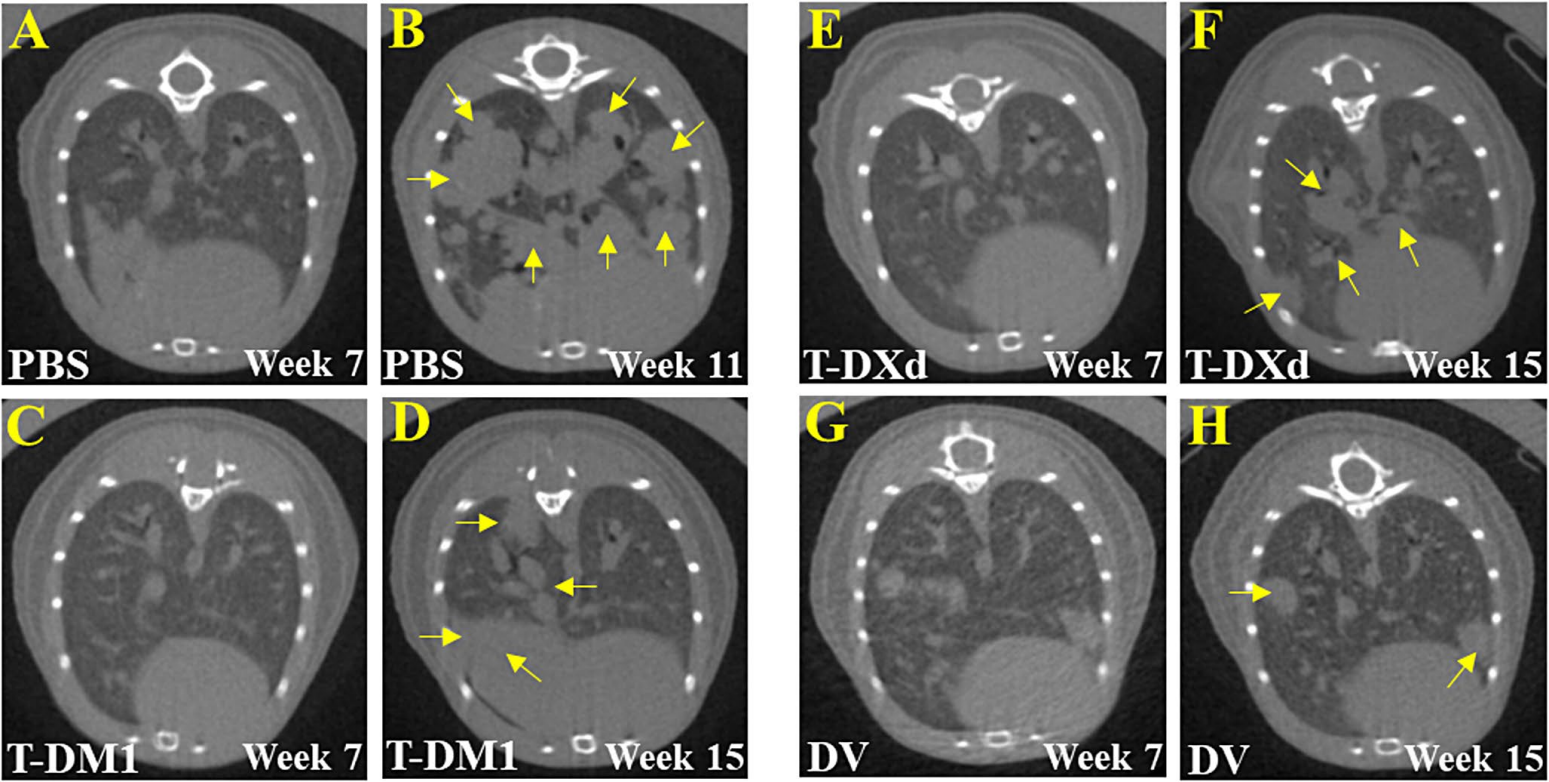
Representative micro-CT images of mice injected with L-JIMT-1 cells intravenously and then treated with PBS (**A**-**B**), T-DM1 (**C**-**D**), T-DXd (**E**-**F**), or DV (**G**-**H**) 8 weeks after the injection. Micro-CT images were taken on week 7 (before treatment) and at the end of the study (PBS treatment, week 11; ADC treatments, week 15). The yellow arrows in panels B, D, F, and H point at lung metastases at the end of the study

All PBS-treated mice were euthanized due to breathing difficulties, weight loss, or deterioration of the body condition score by week 11 or 12, and three T-DM1-treated mice were euthanized on week 14. The remaining mice lived asymptomatic until week 15 when sacrificed for tissue sampling. Mice treated with ADCs lived longer than the PBS-treated mice (*p* ˂ 0.001; Fig. 5B). An example of tumor progression and tumor response are provided in Fig. 6.

After euthanizing the mice, the lungs were removed and fixed. At immunohistochemistry, the number of metastases in the lungs was counted on subserial tissue sections stained for HER2. The number of lung metastases was smaller when the mice were treated with T-DM1, T-DXd, or DV compared to PBS (*p* = 0.04, 0.005, and < 0.001, respectively). The number of lung metastases was lower in the T-DXd-treated and DV-treated mice than in the T-DM1 group mice (*p* = 0.046, and < 0.001, respectively), and smaller in the DV group mice compared to the T-DXd group mice (*p* = 0.021; Fig. 5C). The diameters of the lung metastases were similar in the PBS group and the T-DM1 group (*p* = 0.947), and smaller in the T-DXd and DV groups compared to the T-DM1 group (*p* = 0.002 and < 0.001, respectively). There was no statistical difference in the lung metastasis diameters between the T-DXd group and the DV group (*p* = 0.110; Fig. 5D).

## Discussion

Most patients with HER2-positive metastatic breast cancer eventually progress despite treatments, such as the currently approved anti-HER2 ADCs, T-DM1 and T-DXd, and have limited therapeutic options [5, 18]. HER2-positive breast cancer frequently metastasizes to the lungs [7, 8], but preclinical models for multiresistant lung metastases are lacking, which hampers the development of novel therapeutic agents and strategies for such patients. In this study, we describe a new mouse model for multiresistant HER2-positive breast cancer with lung metastases and compare the anti-cancer effects of three HER2-targeting ADCs both in vitro and in vivo using the model. Disitamab vedotin, a novel HER2-targeting ADC, showed improved efficacy compared to T-DM1 and T-DXd in this model.

Previously reported animal models of HER2-positive human breast cancer lung metastases have been established from the HER2-positive SKBR-3, BT-474, or MDA-MB-453 breast cancer cell lines [19–21]. The SKBR-3 and BT-474 cells are highly sensitive to anti-HER2 drugs including TKIs, monoclonal antibodies, and ADCs, and the MDA-MB-453 cells are partially sensitive to lapatinib and highly sensitive to T-DM1 [10, 16, 17, 22]. In contrast, both the JIMT-1 and the L-JIMT-1 breast cancer cells were resistant to almost all anti-HER2 agents tested and may thus reflect better the clinical scenario with multiresistant disease. In general, the L-JIMT-1 cells were more drug resistant than the parental JIMT-1 cells. Both cell lines were resistant to the EGFR inhibitor erlotinib and the dual EGFR/HER2 inhibitors afatinib, lapatinib, and sapitinib, and the L-JIMT-1 cells were also to the HER2 inhibitor tucatinib. Similarly, while both cell lines were resistant to T-DXd, JIMT-1 cells were more sensitive to T-DM1 and DV than L-JIMT-1 cells. The causes of the greater acquired drug resistance of the L-JIMT-1 cells compared with JIMT-1 cells remain unclear, but, hypothetically, could involve the upregulation of drug efflux pumps that discard toxins from the cells, and up-regulation of p95HER2 expression, the truncated form of HER2 that lacks most of the extracellular domain of the protein [15, 18, 23].

L-JIMT-1 cells formed lung metastases more effectively than JIMT-1 cells and had higher EGFR and HER2 expression than JIMT-1 cells. Both cell lines lacked ER and PgR expression. These observations are well in agreement with a prior observation from a large breast cancer patient cohort suggesting that primary breast cancers with high EGFR and HER2 expression and low PgR expression have a propensity to give rise to lung metastases [7].

All three studied ADCs showed activity in the L-JIMT-1 mouse model, but their efficacy varied. The most active agent in these models was DV both in vitro and in vivo. In vitro, DV was the only ADC that inhibited the growth of L-JIMT-1cells, and it had a stronger inhibitory effect on the JIMT-1 cells than T-DM1. In addition, DV had the strongest inhibitory effect of the three ADCs on the HER2-positive SKBR-3 control cells. In line with these in vitro observations, DV performed well also in the in vivo L-JIMT-1 mouse model inhibiting efficiently metastasis growth, which lead to a smaller number of lung metastases compared with T-DM1 and T-DXd.

There was discrepancy between the in vitro and in vivo activity of T-DXd, since T-DXd had little activity in L-JIMT-1 cells in vitro, but clear activity in vivo in the L-JIMT-1 mouse model. ADCs are composed of three parts, a monoclonal antibody that targets the ADC to cancer cells, a cytotoxic payload intended to kill the targeted cells, and a linker between the two [18, 23]. T-DM1 and T-DXd have the same antibody backbone, trastuzumab, whereas the antibody of DV, hertuzumab, binds to a different epitope on the HER2 receptor [23, 24]. Importantly, both trastuzumab and hertuzumab have anti-tumor effects in vivo on their own, since they can evoke antibody-dependent cellular cytotoxicity (ADCC) through immune effector cells [16, 23–25]. The payloads of T-DM1 and DV, the maytansine derivative DM1 and monomethyl auristatin E (MMAE), respectively, inhibit the microtubule assembly after being internalized and released intracellularly [24, 26], whereas the payload of T-DXd is a DNA topoisomerase I inhibitor exatecan derivative (DXd) [27]. In T-DM1, the payload is conjugated to the monoclonal antibody via a nonreducible thioether linker, whereas T-DXd and DV have an enzymatically cleavable linker [24, 26, 27]. The payloads of T-DXd and DV are also highly membrane-permeable, whereas the active catabolite of DM1, lysine-MCC-DM1, has low membrane permeability. Therefore, the cytotoxic moieties of DV and T-DXd can enter the neighboring cells causing a bystander killing effect, while T-DM1 lacks this effect [28, 29]. ADCC and the bystander effect may at least in part explain why the L-JIMT-1-derived lung metastases were sensitive to T-DXd in vivo despite being resistant in vitro. Similarly, ADCC and the bystander effect could contribute to the anti-cancer efficacy of DV on lung metastases in vivo, and ADCC could also influence the efficacy of T-DM1 in vivo.

The study has some limitations. We did not study in detail the molecular mechanisms that drive the differences in the responsiveness to HER2-targeting agents between the JIMT-1 and the L-JIMT-1 models. These mechanisms remain unknown and warrant future study with e.g. proteomics, phosphoproteomics, and genomics, and by comparisons of the HER2 signaling pathway component activity in the models. Further research is also needed for understanding the molecular mechanisms that explain the propensity of JIMT-1 and L-JIMT-1 cells to give rise to lung metastases. We did not study the activity of T-DM1, T-DXd, and DV in a JIMT-1 mouse model, which is also a topic for further research. Both JIMT-1 and L-JIMT-1 cells are resistant to many targeted agents, whereas the development of resistance to anti-HER2 agents may be gradual in the clinic and involve several molecular mechanisms [30], suggesting that other models are needed for studying this process.

T-DXd and T-DM1 have been approved by the U.S. Food and Drug Administration (FDA) and the European Medicines Agency (EMA) for the treatment of advanced HER2-positive breast cancer [4, 5, 29, 31], whereas DV has not been approved by these agencies. The present results suggest that DV may be a potent drug for the treatment of breast cancer patients with multiresistant HER2-positive disease and support evaluation of DV in clinical trials.

## Conclusions

We developed a novel preclinical human HER2-positive L-JIMT-1 breast cancer lung metastasis mouse model. We compared three HER2-targeting ADCs and five TKIs in this model, and disitamab vedotin turned out to be the most effective agent. The model may assist evaluation of the efficacy of novel and approved agents for multiresistant metastatic HER2-positive breast cancer.

### Electronic supplementary material

Below is the link to the electronic supplementary material.

Supplementary Material 1

Supplementary Material 2

## Data Availability

All data supporting the findings in this study are included within the manuscript and the supplement figures. The raw data used for analysis are available from the corresponding author based on a reasonable request.

## Abbreviations

ADC: antibody-drug conjugate
ADCC: antibody-dependent cellular cytotoxicity
ATCC: American Type Culture Collection
BCS: body condition score
BSA: bovine serum albumin
DMEM: Dulbecco´s modified eagle medium
DM1: derivative of maytansine
DXd: deruxtecan
DV: disitamab vedotin
EGFR: human epidermal growth factor receptor
EMA: European Medicines Agency
ER: estrogen receptor
FBS: fetal bovine serum
FDA: U.S. Food and Drug Administration
HER2: human epidermal growth factor receptor-2
HER3: human epidermal growth factor receptor-3
IC_50_: half-maximal (50%) inhibitory concentration
L-JIMT-1: lung metastatic JIMT-1
PBS: phosphate buffered saline
PgR: progesterone receptor
SCID: severe combined immunodeficient
T-DM1: trastuzumab emtansine
T-DXd: trastuzumab deruxtecan
TKI: tyrosine kinase inhibitor
TVV: tumor and vasculature volume
