# Supplementary material for: Comparison of trastuzumab emtansine, trastuzumab deruxtecan, and disitamab vedotin in a multiresistant HER2-positive breast cancer lung metastasis model
Source: Clin Exp Metastasis. 2024 Feb 17;41(2):91–102. doi: 10.1007/s10585-024-10278-2 (PMC10973002; doi:10.1007/s10585-024-10278-2)
Supplement: Supplementary file 1 — Supplementary Material 1 [file 10585_2024_10278_MOESM1_ESM.docx]

**Supplementary Table 1** Tyrosine kinase inhibitors and antibody drug conjugates studied

| **Drug** | **Drug type** | **Main target receptor** | **Mechanism of action** | **FDA approved indication (year of approval)** |
| --- | --- | --- | --- | --- |
| Afatinib (Gilotrif) (1) | Small molecule | mEGFR  EGFR  HER2 | Tyrosine kinase inhibitor | Advanced non-small cell lung cancer (2013) (1) |
| Erlotinib (Tarceva) (2) | Small molecule | EGFR | Tyrosine kinase inhibitor | Advanced non-small cell lung cancer (2004);  Advanced pancreatic cancer (2005) (2) |
| Lapatinib (Tykerb) (3) | Small molecule | EGFR  HER2 | Tyrosine kinase inhibitor | Advanced HER2+ breast cancer (2007) (3) |
| Sapitinib (AZD8931) (4) | Small molecule | EGFR  HER2 | Tyrosine kinase inhibitor | No |
| Tucatinib (Tuksya) (5) | Small molecule | HER2 | Tyrosine kinase inhibitor | HER2+ advanced breast cancer (2020) (5) |
| Trastuzumab emtansine (T-DM1, Kadcyla) (6) | Antibody-drug conjugate (trastuzumab-SMCC-DM1) | HER2 | Microtubule inhibitor | HER2+ advanced breast cancer (2013) (7);  HER2+ early breast cancer (2019) (8) |
| Trastuzumab deruxtecan (T-DXd, Enhertu) (9) | Antibody-drug conjugate(trastuzumab-MGGPG-exatecan mesylate) | HER2 | Topoisomerase I inhibitor | HER2+ advanced breast cancer (2019) (10);  HER2+ advanced gastric cancer (2021) (11);  HER2-low advanced breast cancer (2022) (12);  HER2-mutated advanced non-small cell lung cancer (2022) (13) |
| Disitamab vedotin (DV, Aidixi) (14, 15, 16) | Antibody-drug conjugate (hertuzumab-MC-Val-Cit-PABC-MMAE) | HER2 | Microtubule inhibitor | Not approved  Conditional NMPA approval for HER2+ advanced gastric cancer (14, 16, 17) and  for HER2+ advanced urothelial cancer in China (2021) (18) |

Abbreviations: DM1, derivative of maytansine 1; EGFR, human epidermal growth factor receptor; FDA, U.S. Food and Drug Administration; HER2, human epidermal growth factor receptor-2; MC-Val-Cit-PABC, maleimidocaproyl-valine-citrulline-p-aminobenzylalcohol-p-nitrophenyl carbonate; mEGFR, mutant human epidermal growth factor receptor; MGGPG, maleimide glycine-glicyne-phenylalanine-glycine; MMAE, monomethyl auristatin E; NMPA, National Medical Products Administration of China; SMCC, N-succinimidyl-4-(N-maleimidomethyl) cyclohexane-1-carboxylate

**References**

1. Dungo RT, Keating GM. Afatinib: first global approval. Drugs. 2013;73(13):1503-15.

2. Bareschino MA, Schettino C, Troiani T, Martinelli E, Morgillo F, Ciardiello F. Erlotinib in cancer treatment. Ann Oncol. 2007;18 Suppl 6:vi35-41.

3. Ryan Q, Ibrahim A, Cohen MH, Johnson J, Ko CW, Sridhara R, et al. FDA drug approval summary: lapatinib in combination with capecitabine for previously treated metastatic breast cancer that overexpresses HER-2. Oncologist. 2008;13(10):1114-9.

4. Morrison G, Fu X, Shea M, Nanda S, Giuliano M, Wang T, et al. Therapeutic potential of the dual EGFR/HER2 inhibitor AZD8931 in circumventing endocrine resistance. Breast Cancer Res Treat. 2014;144(2):263-72.

5. Shah M, Wedam S, Cheng J, Fiero MH, Xia H, Li F, et al. FDA Approval Summary: Tucatinib for the Treatment of Patients with Advanced or Metastatic HER2-positive Breast Cancer. Clin Cancer Res. 2021;27(5):1220-6.

6. Lewis Phillips GD, Li G, Dugger DL, Crocker LM, Parsons KL, Mai E, et al. Targeting HER2-positive breast cancer with trastuzumab-DM1, an antibody-cytotoxic drug conjugate. Cancer Res. 2008;68(22):9280-90.

7. Verma S, Miles D, Gianni L, Krop I, Welslau M, Baselga J, et al. Trastuzumab emtansine for HER2-positive advanced breast cancer. N Engl J Med. 2012;367:1783 - 91.

8. von Minckwitz G, Huang CS, Mano MS, Loibl S, Mamounas EP, Untch M, et al. Trastuzumab Emtansine for Residual Invasive HER2-Positive Breast Cancer. N Engl J Med. 2018.

9. Ogitani Y, Hagihara K, Oitate M, Naito H, Agatsuma T. Bystander killing effect of DS-8201a, a novel anti-human epidermal growth factor receptor 2 antibody-drug conjugate, in tumors with human epidermal growth factor receptor 2 heterogeneity. Cancer Sci. 2016;107(7):1039-46.

10. Modi S, Saura C, Yamashita T, Park YH, Kim SB, Tamura K, et al. Trastuzumab Deruxtecan in Previously Treated HER2-Positive Breast Cancer. N Engl J Med. 2020;382(7):610-21.

11. Shitara K, Bang YJ, Iwasa S, Sugimoto N, Ryu MH, Sakai D, et al. Trastuzumab Deruxtecan in Previously Treated HER2-Positive Gastric Cancer. N Engl J Med. 2020;382(25):2419-30.

12. Modi S, Jacot W, Yamashita T, Sohn J, Vidal M, Tokunaga E, et al. Trastuzumab Deruxtecan in Previously Treated HER2-Low Advanced Breast Cancer. N Engl J Med. 2022;387(1):9-20.

13. Li BT, Smit EF, Goto Y, Nakagawa K, Udagawa H, Mazieres J, et al. Trastuzumab Deruxtecan in HER2-Mutant Non-Small-Cell Lung Cancer. N Engl J Med. 2022;386(3):241-51.

14. Jiang J, Li S, Shan X, Wang L, Ma J, Huang M, et al. Preclinical safety profile of disitamab vedotin：a novel anti-HER2 antibody conjugated with MMAE. Toxicol Lett. 2020;324:30-7.

15. Li H, Yu C, Jiang J, Huang C, Yao X, Xu Q, et al. An anti-HER2 antibody conjugated with monomethyl auristatin E is highly effective in HER2-positive human gastric cancer. Cancer Biol Ther. 2016;17(4):346-54.

16. Deeks ED. Disitamab Vedotin: First Approval. Drugs. 2021;81(16):1929-35.

17. Peng Z, Liu T, Wei J, Wang A, He Y, Yang L, et al. Efficacy and safety of a novel anti-HER2 therapeutic antibody RC48 in patients with HER2-overexpressing, locally advanced or metastatic gastric or gastroesophageal junction cancer: a single-arm phase II study. Cancer Commun (Lond). 2021;41(11):1173-82.

18. Fu Z, Li S, Han S, Shi C, Zhang Y. Antibody drug conjugate: the "biological missile" for targeted cancer therapy. Signal Transduct Target Ther. 2022;7(1):93.
