# Supplementary material for: Comparison of trastuzumab emtansine, trastuzumab deruxtecan, and disitamab vedotin in a multiresistant HER2-positive breast cancer lung metastasis model
Source: Clin Exp Metastasis. 2024 Feb 17;41(2):91–102. doi: 10.1007/s10585-024-10278-2 (PMC10973002; doi:10.1007/s10585-024-10278-2)
Supplement: Supplementary file 2 — Supplementary Material 2 [file 10585_2024_10278_MOESM2_ESM.docx]

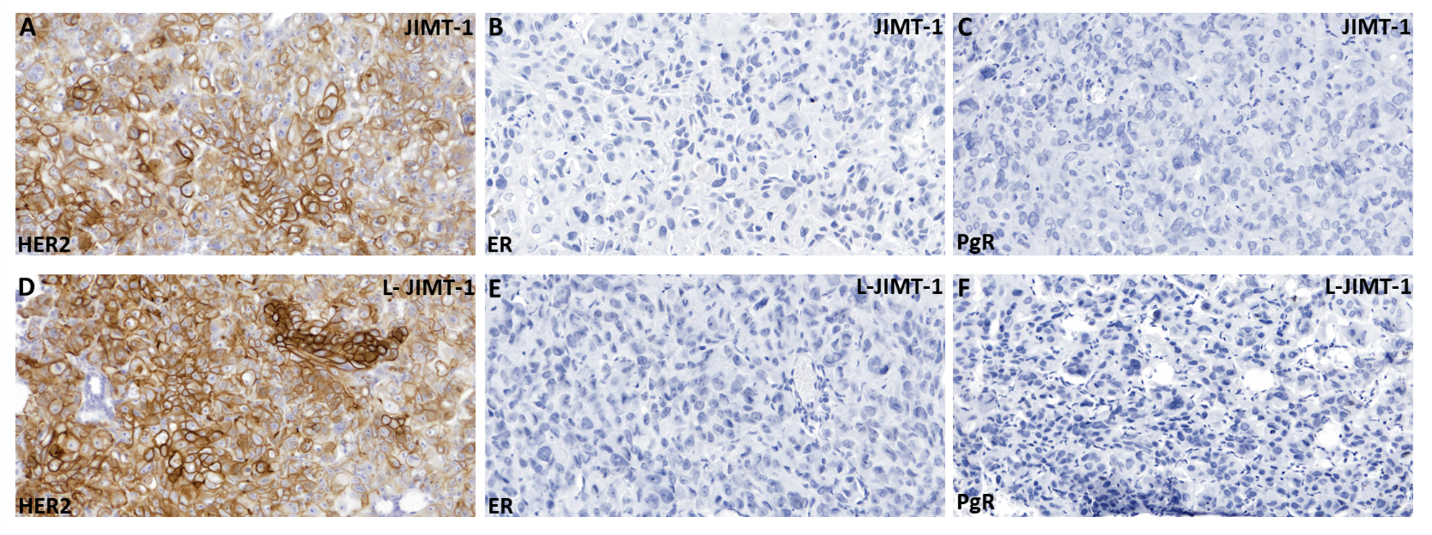


**Supplementary Fig. 1.** Examples of JIMT-1 derived (A-C) and L-JIMT-1 derived (D-F) lung metastases stained with immunohistochemistry. HER2, human epidermal growth factor receptor 2; ER, estrogen receptor; PgR, progesterone receptor.
